# Impact of Saroglitazar on Liver Stiffness Measurements in Metabolic Dysfunction-Associated Steatotic Liver Disease (MASLD) With Compensated Cirrhosis: A Single-Arm Study

**DOI:** 10.7759/cureus.92313

**Published:** 2025-09-14

**Authors:** Akash Roy, Shardhya Chakraborty, Surabhi Jajodia, Usha Goenka, Awanish Tewari, Nikhil Sonthalia, Uday C Ghoshal, Mahesh Goenka

**Affiliations:** 1 Gastroenterology, Apollo Multispeciality Hospitals Kolkata, Kolkata, IND; 2 Dietetics, Apollo Multispeciality Hospitals Kolkata, Kolkata, IND; 3 Clinical Imaging and Interventional Radiology, Apollo Multispeciality Hospitals Kolkata, Kolkata, IND

**Keywords:** cirrhosis, elastography, liver fat content, masld, saroglitazar

## Abstract

Background

Saroglitazar, a dual peroxisome proliferator-activated receptor α/γ agonist, shows promise in metabolic dysfunction-associated steatotic liver disease (MASLD). However, its impact and safety in MASLD with compensated cirrhosis (MASLD-CC) remain relatively unexplored.

Methods

In a single-center, single-arm retrospective study, we assessed the impact of Saroglitazar (4 mg for 24 weeks) on liver stiffness measurement (LSM) by magnetic resonance elastography (MRE) in MASLD-CC and evaluated safety and effects on biochemical indices and liver fat content (LFC) by MRI-proton density fat fraction (MRI-PDFF). To distinguish measurement error from true change, a threshold of 0.75 kPa for MRE-LSM was used to categorize LSM progressors and regressors.

Results

Twenty-six patients with MASLD-CC (age 61 (58-64) years; 61.5% female; BMI 28.7 ± 3.3 kg/m²; 88.4% obese; 76.9% with diabetes) completed 24 weeks of follow-up. Body weight (69.5 (64-75) vs 68.3 (66-71) kg, p = 0.02), alanine transaminase (42.5 (30-61) vs 22.5 (20-36) U/L), aspartate transaminase (53 (39-74) vs 40 (33-49) U/L), fasting blood sugar (109 (100-129.5) vs 100.5 (96.3-108.3) mg/dL), total cholesterol (157 (139.5-171) vs 140 (120-167) mg/dL), triglycerides (121 (95-153) vs 100 (94-113.5) mg/dL), and LFC (7.2 (6.9-11.1) vs 5.1 (3.5-6.1) %) decreased with Saroglitazar (p < 0.01 for all). A decrease in MRE-LSM (5.4 (4.9-6.2) vs 5.1 (4.9-5.8) kPa) was seen in the overall cohort (p = 0.04). However, only 4 (15.3%) and 2 (7.6%) showed MRE-LSM regression and progression of ≥ 0.75 kPa, respectively. No differences were noted between regressors and progressors using the 0.75 kPa threshold (23.5% vs 22.2%, p = 0.91). Two patients (pruritus and increased urine frequency) reported adverse events.

Conclusion

Saroglitazar use over 24 weeks in MASLD-CC appears safe. Using pragmatic thresholds, changes in liver stiffness over 24 weeks did not differ significantly. Larger prospective studies with longer follow-up are warranted.

## Introduction

Metabolic dysfunction-associated steatotic liver disease (MASLD) has emerged as the leading cause of chronic liver disease globally, affecting more than a third of the world’s adult population [1]. The burden of MASLD is set to increase exponentially worldwide, with a projected rise of 63% between 2015 and 2030 [2]. While such a large population is at risk for MASLD as a whole, the presence of advanced fibrosis or cirrhosis remains the chief determinant of primary adverse liver outcomes (MALO) [3]. Approximately 10-25% of patients with MASLD-related steatohepatitis develop advanced fibrosis and cirrhosis [4]. Recent population-based data show the prevalence of advanced fibrosis and cirrhosis due to MASLD in 8.1% and 1.1% of the population, respectively [5].

In contrast to the meteoric rise in disease burden, the therapeutic armamentarium against MASLD remains limited. In the past, treatment strategies were limited to risk-factor mitigation, lifestyle and dietary adjustments, and vitamin E [6]. Recently, resmetirom, a thyroid hormone receptor-β agonist, was approved as the first drug for treating patients with noncirrhotic metabolic dysfunction-associated steatohepatitis (MASH) [7,8]. However, it is currently not available in many parts of the world, including Asia, and is not recommended for those with cirrhosis [9,10]. Hence, therapeutic options for this population remain limited.

Saroglitazar is a peroxisome proliferator-activated receptor (PPAR) agonist, granted marketing authorization in India in 2013 for the management of atherogenic diabetic dyslipidemia and subsequently endorsed for the treatment of MASLD [11,12]. Two systematic reviews and one randomized trial have demonstrated its efficacy in improving non-invasive liver disease assessment (NILDA) parameters of liver fibrosis and steatosis [13-15]. Overall, it has shown an excellent safety profile [13-15]. However, the safety and effects of saroglitazar are relatively unknown in those with compensated cirrhosis, with only a subgroup of a single study (n = 11) having F4 fibrosis based on transient elastography [16]. In this context, we aimed to assess the safety and effects of saroglitazar using magnetic resonance elastography (MRE) and MRI-proton density fat fraction (MRI-PDFF) as NILDA measures.

This article was previously presented as a meeting abstract at the Liver Meeting (AASLD 2024), San Diego, USA.

## Materials and methods

Study setting and design

This was a retrospective analysis from a prospectively maintained cohort of patients with MASLD-related compensated cirrhosis attending a dedicated liver clinic in a tertiary-care hospital in Eastern India.

Inclusion criteria

The study population comprised individuals aged ≥ 18 years who met the currently proposed criteria for MASLD (satisfying at least one of five cardiometabolic criteria) and had compensated cirrhosis defined by radiological or histological features, or endoscopic features of portal hypertension, with a contemporaneous MRE liver stiffness measurement (MRE-LSM) suggestive of at least F4 fibrosis, defined as ≥ 4.45 kPa, as per previously published literature [17,18]. All patients were assessed for the presence of compensated cirrhosis at baseline by an experienced hepatologist/gastroenterologist.

Exclusion criteria

Patients with a current or past history of decompensated cirrhosis (ascites, hepatic encephalopathy, variceal bleeding, or a Child-Turcotte-Pugh score ≥ 7); other causes of chronic liver disease (except MASLD) or secondary causes of steatotic liver disease; a history of significant alcohol consumption; total serum bilirubin > 2.5 mg/dL; international normalized ratio (INR) > 1.7; hepatocellular carcinoma; concomitant diseases with reduced life expectancy (≤ 6 months); chronic kidney disease (stage III or above); known HIV infection; type 1 diabetes; inability to undergo MRI or inability to generate validated MRE due to technical issues; congestive cardiac failure; cholestatic liver disease; or iron-overload states were excluded [19,20]. Individuals with a known allergy or intolerance to saroglitazar; women who were pregnant or lactating or of childbearing potential and not using appropriate contraceptive measures; and those who had used vitamin E in any formulation or dose within 1 month preceding the screening visit were also excluded.

Patient data

Baseline demographic, clinical, and laboratory data were collected at enrollment, including age, sex, body weight (kg), height, BMI, comorbidities, and self-reported alcohol consumption. Laboratory parameters included complete blood count, liver and renal function tests, lipid profile, and fasting blood glucose.

MRE technique

MRIs were obtained using a 3.0-T device (Philips; spin-echo echo-planar imaging protocol; manufacturer: Philips, Tokyo, Japan). Once the patient was on the examination table, a pneumatic driver was placed over the upper abdomen to generate acoustic mechanical waves to the liver. The operating frequency of the driver was approximately 60 Hz. MR elastography SE-EPI sequence was obtained using a body coil with the following parameters: TR/TE 1066/58 ms, flip angle 90°, field of view 40-40-8.7 cm, matrix 256 × 64, thickness 10 mm. Once the images were acquired, the data were automatically post-processed to generate a stiffness map. Stiffness was measured in kPa as the mean value from eight representative regions of interest across eight separate slices.

Follow-up

Saroglitazar 4 mg was prescribed once daily to eligible patients, and follow-up began on the date of initiation. Treatment was evaluated in person or by telephone at regular intervals for any adverse events. Laboratory indices, MRI-PDFF, and MRE were repeated at the end of 6 months or sooner if any clinical deterioration or change in symptoms occurred. Patients who did not have two available MREs were excluded.

Outcome measures

The primary outcome measure was change in liver stiffness (MRE-LSM).

Other outcome measures included safety, changes in liver fat content (LFC) by MRI-PDFF, and biochemical indices.

Exploratory Outcome Measure

Because elastography measures have repeatability variation that may not translate into true biological change, a threshold of 0.75 kPa has been suggested for MRE-LSM measurements [21]. Accordingly, to minimize measurement error, we classified patients as MRE regressors if they had a decline of ≥ 0.75 kPa or MRE progressors if they had an increase of ≥ 0.75 kPa.

Sample size calculation and statistical analysis

The only available study in patients with cirrhosis showed a mean difference in liver stiffness of 5.75 kPa and a percentage difference of 22.9% pre- to post-saroglitazar treatment in 11 patients. Based on this, a minimum of 23 paired observations was required to achieve 80% power at a two-sided 5% level of significance [16]. The Kolmogorov-Smirnov test was used to assess normality. Descriptive statistics were reported as numbers (%), mean ± SD, or median (range), as appropriate. Categorical variables were compared using Pearson’s chi-squared test. Normally distributed continuous data were analyzed using paired t tests. The Wilcoxon signed-rank test was used for skewed, non-normal data. A p-value < 0.05 was considered statistically significant.

Ethics

The institutional review board gave ethical approval for the study (IEC/BR/03/12). All research procedures were conducted in accordance with the principles outlined in the 1975 Declaration of Helsinki.

## Results

A total of 26 individuals (median age 61.5 years (58-64); 61.5% female; BMI 28.7 ± 3.3 kg/m²; 88.4% obese; 76.9% with diabetes) with MASLD cirrhosis had available data at 24 weeks, with at least two MRE readings pre- and post-saroglitazar therapy. The baseline characteristics are shown in Table 1.

**Table 1:** Baseline characteristics of individuals with MASLD and compensated cirrhosis. Data are expressed as median (IQR). MRE: Magnetic resonance elastography; MASLD: Metabolic dysfunction-associated steatotic liver disease.

Characteristics	Value (n = 26)
Female (%)	18 (69.2)
Male (%)	8 (30.8)
Age (years)	61 (58-64)
BMI (kg/m²)	28.7 ± 3.3
Weight (kg)	69.5 (64-75)
Waist circumference (cm)	98.8 ± 6.9
Obese (%)	23 (88.4)
Diabetes (%)	20 (76.9)
Hypertension (%)	16 (61.5)
Dyslipidemia (%)	9 (34.6)
Hemoglobin (g/dL)	11.5 (10.6-12.3)
Total leukocyte count (×10³/mm³)	6.4 ± 2.0
Platelet count (×10³/mm³)	146 (126-160)
Total bilirubin (mg/dL)	0.9 (0.7-1.0)
Alanine transaminase (IU/L)	42.5 (30-61)
Aspartate transaminase (IU/L)	53 (39-74)
Alkaline phosphatase (IU/L)	98.5 (80-152)
Fasting blood sugar (mg/dL)	109 (100-129.5)
Total cholesterol (mg/dL)	157 (139.5-171)
Low-density lipoprotein (mg/dL)	94 (76-107)
High-density lipoprotein (mg/dL)	42 (36.5-43)
Triglycerides (mg/dL)	121 (95-153)
MRI-proton density fat fraction (%)	7.9 (6.9-11.6)
MRE-liver stiffness measurement (kPa)	5.4 (4.9-6.2)

Change in clinical and biochemical parameters at 24 weeks

At 24 weeks, there was a decrease in body weight from 69.5 (64-75) kg to 68.3 (66-71) kg (p = 0.02). There were no significant changes in serum bilirubin (0.9 (0.7-1.0) vs 1.0 (0.6-1.1) mg/dL, p = 0.70). Significant changes were noted in alanine transaminase (42.5 (30-61) vs 22.5 (20-36) U/L) and aspartate transaminase (53 (39-74) vs 40 (33-49) U/L), as well as fasting blood sugar and lipid profile parameters (p < 0.01 for all) (Table 2).

**Table 2:** Changes in clinical, biochemical, liver fat content, and elastography measures with Saroglitazar. Data are expressed as median (IQR). Wilcoxon signed-rank test. MRE: Magnetic resonance elastography.

Characteristics	Pretreatment	Post-treatment	p-value
Weight (kg)	69.5 (64-75)	68.3 (66-71)	0.02
Total bilirubin (mg/dL)	0.9 (0.7-1.0)	1.0 (0.6-1.1)	0.7
Alanine transaminase (IU/L)	42.5 (30-61)	22.5 (20-36)	<0.01
Aspartate transaminase (IU/L)	53 (39-74)	40 (33-49)	<0.01
Alkaline phosphatase (IU/L)	98.5 (80-152)	86.5 (62-101)	0.01
Fasting blood sugar (mg/dL)	109 (100-129.5)	100.5 (96.3-108.3)	<0.01
Total cholesterol (mg/dL)	157 (139.5-171)	140 (120-167)	<0.01
Low-density lipoprotein (mg/dL)	94 (76-107)	89 (79.5-100)	0.33
High-density lipoprotein (mg/dL)	42 (36.5-43)	40 (36.5-47)	0.39
Triglycerides (mg/dL)	121 (95-153)	100 (94-113.5)	<0.01
MRI-proton density fat fraction (%)	7.9 (6.9-11.6)	5.2 (3.3-6.3)	<0.01
MRE-liver stiffness measurement (kPa)	5.4 (4.9-6.2)	5.1 (4.9-5.8)	0.04

Changes in LFC on MRI-PDFF and MRE-LSM

**Figure 1:**
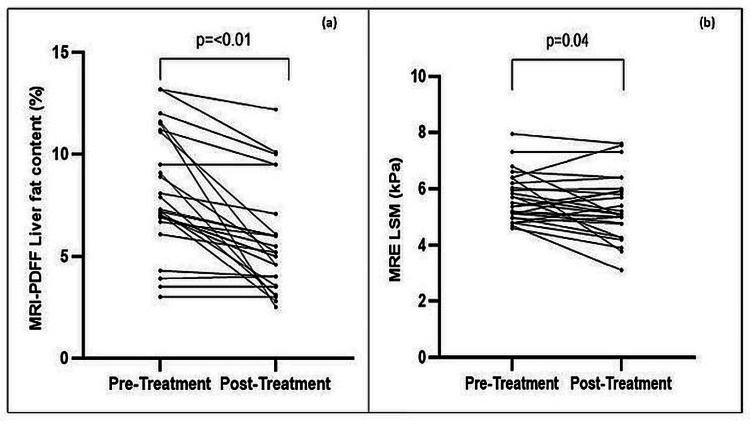
(a) Changes in liver fat content by MRI-PDFF; (b) Changes in liver stiffness measurement by MRE. PDFF: Proton density fat fraction; MRE: Magnetic resonance elastography. Test of significance: Wilcoxon signed-rank test.

LFC on MRI-PDFF decreased from 7.9 (6.9-11.6)% to 5.2 (3.3-6.3)% (p < 0.01) at 24 weeks in the whole cohort. Overall, MRE-LSM decreased from 5.4 (4.9-6.2) kPa to 5.1 (4.9-5.8) kPa (p = 0.04). The results of changes in MRI-PDFF and MRE-LSM are shown in Table 2 and Figure 1.

Progressors and regressors on MRE-LSM

As noted earlier, identifying true progressors and regressors is important in studies using NILDA measures. A total of 17 patients showed any MRE-LSM regression, of whom only 4 (23.5%) exhibited a regression of ≥ 0.75 kPa. Conversely, nine patients showed any progression in MRE-LSM, with only two experiencing a progression of ≥ 0.75 kPa. No differences were noted between regressors and progressors using the 0.75 kPa threshold (23.5% vs 22.2%, p = 0.91) (Table 3).

**Table 3:** Distribution of elastography progressors and regressors. MRE: Magnetic resonance elastography; kPa: Kilopascal.

Parameter	n/total (%)
MRE significant regression (≥ 0.75 kPa decrease) / overall regression (any decrease)	4/17 (23.5%)
MRE significant progression (≥ 0.75 kPa increase) / overall progression (any increase)	2/9 (22.2%)

Adverse events

No significant adverse events were noted in the patients analyzed at follow-up. Adverse events were recorded as self-reporting. Two patients (pruritus and increased urine frequency) reported adverse events that did not require medication discontinuation.

## Discussion

**Figure 2:**
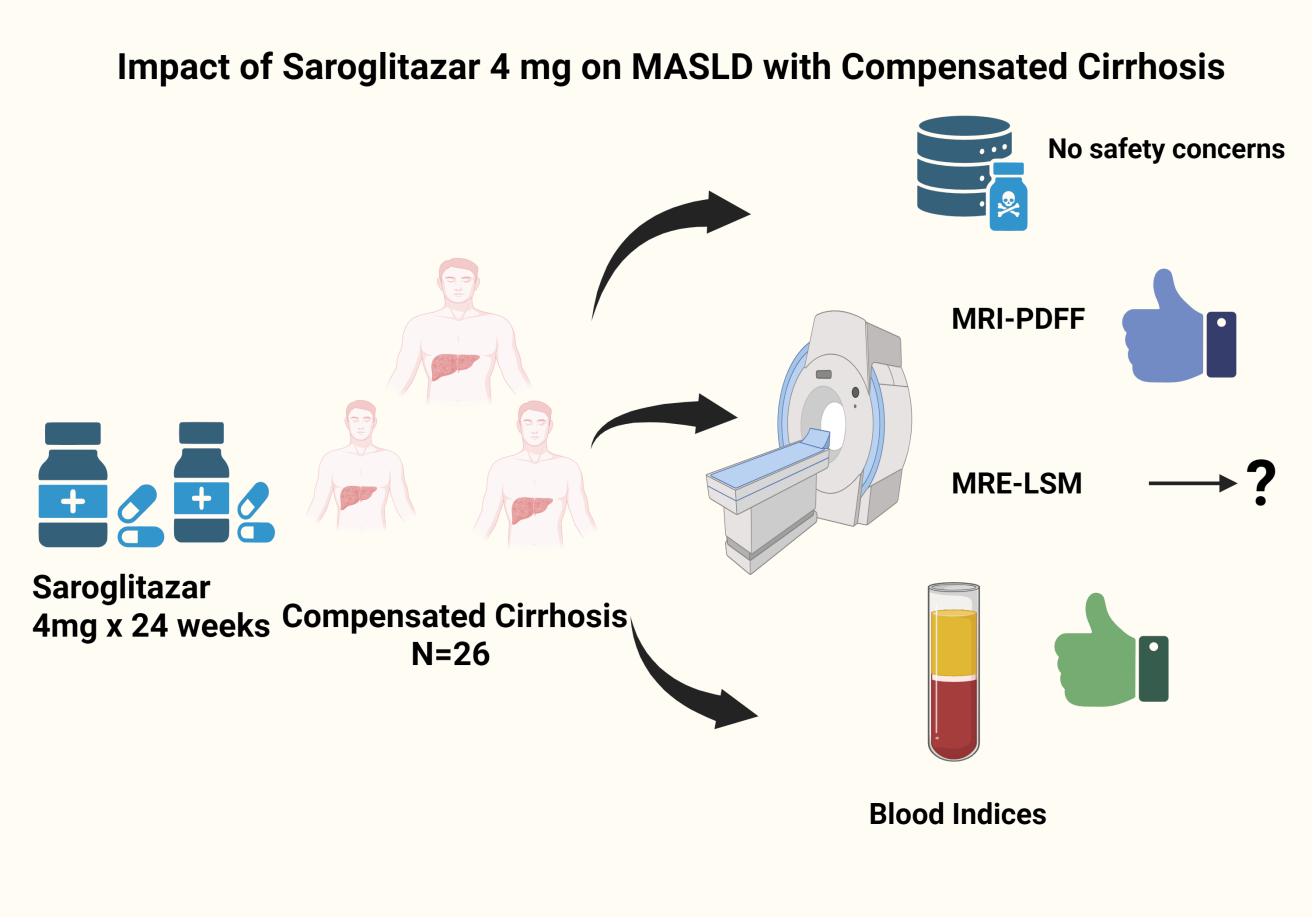
Overall summary of the impact of saroglitazar in compensated cirrhosis. Image credit: Figure is the authors’ own creation; created by A. Roy with BioRender.com. PDFF: Proton density fat fraction; MRE: Magnetic resonance elastography; LSM: Liver stiffness measurement.

In this single-arm study, we evaluated patients with compensated cirrhosis who received 24 weeks of Saroglitazar to assess changes in liver stiffness measurement, liver fat content, and biochemical indices. We found significant improvements in these measures. Saroglitazar was not associated with significant adverse events in this population. However, when analyzing exploratory pragmatic differences using a 0.75 kPa absolute minimum difference, the changes were not significant (Figure 2).

Options for therapy in individuals with compensated MASLD cirrhosis are limited, and management is directed toward the prevention of first decompensation. A single prior retrospective study showed that the use of vitamin E was associated with improved clinical outcomes in patients with MASLD and bridging fibrosis or cirrhosis [19]. Saroglitazar belongs to the class of drugs known as peroxisome proliferator-activated receptor (PPAR) agonists and was introduced primarily for the management of atherogenic diabetic dyslipidemia. Through its dual metabolic, anti-inflammatory, and potential antifibrotic actions, Saroglitazar has emerged as a promising agent for patients with liver disease. Multiple subsequent studies have demonstrated its beneficial effects in reducing aminotransferase levels, glycemic indices, lipid parameters, liver fat content, and liver stiffness (as measured by transient elastography) in individuals with non-cirrhotic MASLD [15,20-23]. A meta-analysis of 10 studies showed significant improvement in liver stiffness in predominantly non-cirrhotic MASLD (mean difference: 2.22 kPa (95% CI: 0.80 to 3.63); p = 0.002; I² = 99%), and another systematic review showed an overall pooled standardized difference in means of LSM reduction of -0.98 (95% CI: -1.1 to -0.8) [13,14]. However, the majority of the studies excluded patients with compensated cirrhosis, except for a subgroup (n = 11) in a single study in which a significant improvement was shown from a baseline LSM of 25.05 ± 6.38 kPa to 19.30 ± 9.06 kPa in those with F4 fibrosis or cirrhosis based on transient elastography. In this context, the current study provides evidence for the use of Saroglitazar in compensated cirrhosis.

While liver biopsy remains the gold standard, its applicability for repeated measures is limited in real-world practice. Liver stiffness assessment has emerged as a robust tool for the diagnosis and staging of hepatic fibrosis [24,25]. Beyond diagnosis and staging, dynamic LSM assessment is also a key prognostic tool, particularly in patients with compensated advanced chronic liver disease (cACLD). A recent study involving 2,508 patients with 8,561 reliable LSMs found that a 20% increase in LSM at any time was associated with an approximately 50% increased risk of hepatic decompensation. Conversely, any LSM decrease to < 20 kPa identified patients with cACLD who had a substantially lower risk of hepatic decompensation [26]. Similarly, an MRE-based study in MASLD showed that among those with compensated cirrhosis at baseline (n = 29), decompensation or death occurred in 100% of LSM progressors and only 19% of non-progressors (p < 0.001) over a median follow-up of 2.5 years [27]. In this context, the current data on LSM progressors and regressors on Saroglitazar, and subsequent follow-up for MALOs, appear promising for future studies. A recent study has also shown the promise of Saroglitazar in Met-ALD, further generating interest in future studies of Saroglitazar [28].

The study did not raise concerns about the use of Saroglitazar for any significant adverse events. Most previous studies with Saroglitazar have shown the molecule to be safe. The safety of Saroglitazar in patients with compensated cirrhosis opens opportunities to assess its effects in this population on a long-term basis, either alone or with vitamin E.

Limitations

The current study has several limitations arising from its exploratory pilot design. Most importantly, the study is retrospective and not adequately powered due to the small sample size, and the results on progression and regression should not be viewed as definitive. Inherent limitations include biases arising from follow-up in a retrospective design. Future larger studies may provide more granular insights. Second, although the current study included compensated cirrhosis, it would be important to analyze the findings in a broader cohort of cACLD. Third, we did not have a historical cohort or concomitant transient elastography results, which would have added further merit. A follow-up period of six months may be short, and longer outcome studies are warranted. Lastly, the absence of histology remains a major limitation.

## Conclusions

Treatment options for compensated cirrhosis are limited. The use of saroglitazar over 24 weeks in MASLD-compensated cirrhosis appears safe, but its impact on long-term clinical outcomes needs to be evaluated. Changes in LSM based on pragmatic endpoints were not statistically significant and should be explored in larger studies.
